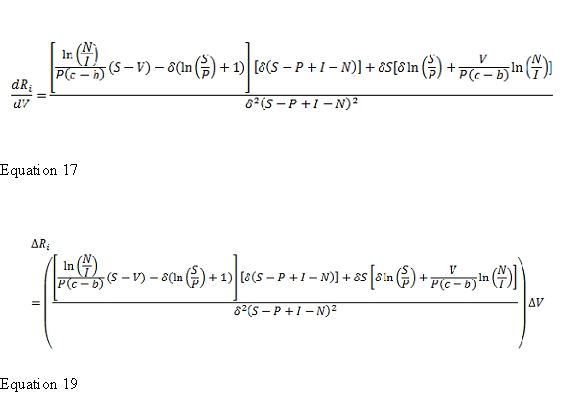# Correction: Predicting the Herd Immunity Threshold during an Outbreak: A Recursive Approach

**DOI:** 10.1371/annotation/01641ef8-cbe2-4ca7-900a-e1d12bd8557a

**Published:** 2009-02-24

**Authors:** Nathan T. Georgette

Equations 17 and 19 appear incorrectly. The corrected Equations 17 and 19 can be found here: